# Calcite incorporated in silica/collagen xerogels mediates calcium release and enhances osteoblast proliferation and differentiation

**DOI:** 10.1038/s41598-019-56023-8

**Published:** 2020-01-10

**Authors:** S. Rößler, R. Unbehau, T. Gemming, B. Kruppke, H.-P. Wiesmann, T. Hanke

**Affiliations:** 1 Max Bergmann Center of Biomaterials and Institute of Materials Science, Technical University Dresden, Budapester Str. 27, D-01069 Dresden, Germany; 2 IFW Dresden, P.O. Box 270116, D-01171 Dresden, Germany; 3 Institute of Materials Research, Helmholtz-Zentrum Geesthacht, Postfach 1160, D-21494 Geesthacht, Germany

**Keywords:** Cell biology, Biomedical engineering, Biomaterials

## Abstract

Multiphasic silica/collagen xerogels are biomaterials designed for bone regeneration. Biphasic silica/collagen xerogels (B30) and triphasic xerogels (B30H20 or B30CK20) additionally containing hydroxyapatite or calcite were demonstrated to exhibit several structural levels. On the first level, low fibrillar collagen serves as template for silica nanoparticle agglomerates. On second level, this silica-enriched matrix phase is fiber-reinforced by collagen fibrils. In case of hydroxyapatite incorporation in B30H20, resulting xerogels exhibit a hydroxyapatite-enriched phase consisting of hydroxyapatite particle agglomerates next to silica and low fibrillar collagen. Calcite in B30CK20 is incorporated as single non-agglomerated crystal into the silica/collagen matrix phase with embedded collagen fibrils. Both the structure of multiphasic xerogels and the manner of hydroxyapatite or calcite incorporation have an influence on the release of calcium from the xerogels. B30CK20 released a significantly higher amount of calcium into a calcium-free solution over a three-week period than B30H20. In calcium containing incubation media, all xerogels caused a decrease in calcium concentration as a result of their bioactivity, which was superimposed by the calcium release for B30CK20 and B30H20. Proliferation of human bone marrow stromal cells in direct contact to the materials was enhanced on B30CK20 compared to cells on both plain B30 and B30H20.

## Introduction

The development of substitute materials for the restore and healing promotion of lost or wounded tissue is associated to a multiplicity of scientific and technical challenges. To match these requests, a lot of materials suitable for bone replacement are inspired by natural tissue^1^. Such materials aim to meet the mechanical requirements in bone relevant ranges, to be bioactive and resorbable, as well as to play an active role in the bone remodeling process^2–4^. The most appropriate inspiration for bone substitute materials is natural bone itself. Other sources of inspiration can be found in marine organisms such as marine glass sponge spicules, the endoskeleton of sea urchins or mollusk shells. All examples have one thing in common, they exhibit an organic matrix that is reinforced by inorganic minerals^4,5^. In mammalian bone, a collagenous matrix is a template that is mineralized by bone apatite^6^. In marine glass sponge spicules, organic molecules act as templates for either silica or calcium carbonate (calcite) mineralization^7,8^. The organic matrix of mollusk shell is also mineralized by calcium carbonate (aragonite)^9^. Bio-inspired materials have huge potential as materials for hard tissue replacement. This is not only true for collagen and hydroxyapatite, but also for collagen and silica or calcium carbonate, respectively, as bi- or triphasic composites.

Osteoporosis is an important and widely distributed alteration of the bone. Osteoporotic bone is characterized by a lower bone mineral density compared to healthy bone resulting in a higher risk for fracturing^10–13^. This symptoms are due to a mismatch between bone resorption and formation of new bone^14,15^. Recent strategies in biomaterial-related support of the healing of fractured osteoporotic bones are focusing on the bone remodeling process in such a way that osteoblastogenesis is supported, while osteoclast activity is not suppressed at all^16^. Hence, the priority function of the substitute material is the enhancement of osteoblast proliferation and differentiation, what we also focus on in the present study.

Extracellular calcium plays an important role in bone metabolism. Usually, studies on the effect of calcium ions were performed for elevated calcium levels compared to the usual concentration in cell culture medium (1.8 mM). An elevated calcium concentration has stimulating effects on osteoblasts and their progenitor cells^17–19^. Moreover, as a result of increased extracellular calcium concentrations various osteoblastic markers such as alkaline phosphatase (ALP), osteocalcin, osteopontin and bone sialoprotein were significantly increased^17,20,21^. Furthermore, the extracellular calcium concentration affects the formation of a mineralized matrix^21,22^. The mechanisms related to the interaction of osteoblasts and their progenitor cells with extracellular calcium are still not fully understood. The interaction based on the activation of the calcium sensing receptor (CaSR) and voltage-dependent calcium ion channels that regulate the maintenance of intracellular calcium homeostasis have been proposed by numerous authors^15,17–19,21,23,24^.

The present study is focused on bio-inspired composites based on silica and collagen as biphasic materials as well as triphasic composites based on silica, collagen and a further mineral phase. These composite materials for bone substitution are inspired by marine glass sponge spicules due to their excellent mechanical properties^7^. To produce multiphasic silica/collagen xerogels, fibrillar bovine collagen was used as template for silica mineralization. Our previous study, published by Heinemann *et al*., focused on the adjustment of mechanical properties in bone relevant ranges by variation of the xerogel composition^25^. Another important property of bi- and triphasic silica/collagen xerogels is their bioactivity^26^. Bioactivity, known from bioglasses or glass ceramics, is a property characterizing the ability of a material to form an apatite layer on its surface when incubated in a calcium and phosphate ion containing environment^27^. Therefore, the ambient liquid becomes depleted from these ions. The addition of different calcium phosphates as a third phase in silica/collagen xerogels was shown to increase bioactivity and thus to be a tool for manipulating the ratio of osteoblasts to osteoclasts^21,28,29^. Previous studies have shown that calcium ion depletion in extracellular space reduces proliferation and osteogenic differentiation of human bone marrow stromal cells (hMSC)^21,30^. Thus, the control of environmental calcium concentration effected by the implanted biomaterial is a promising approach in the perspective treatment of bone defects and fractures, especially in systemically altered bones.

Therefore, biomaterials with the capacity to release calcium ions are promising in the treatment of bone defects. To fulfill the aim of calcium release, calcium carbonate might be a suitable candidate because of its solubility. Ceramics made of calcium carbonate have already been successfully prepared despite of the difficulty that calcium carbonate decomposes to carbon dioxide and calcium oxide above 900 K^31^. Calcium carbonate is biocompatible and has a higher degradation rate than calcium phosphates^32,33^. He *et al*. demonstrated a faster degradation for a calcium carbonate ceramic compared to a biphasic calcium phosphate ceramic *in vitro* and *in vivo*^34^. A time-dependent increase in proliferation and osteogenic differentiation of bone derived cells cultured on porous calcium carbonate (calcite) scaffolds was published by Chróścicka *et al*. and Woldetsadik *et al*.^35,36^. In contrast to calcium carbonate ceramics, the present study focuses on composite materials based on silica, collagen and calcite as a further mineral phase.

In our previous study, multiphasic silica/collagen xerogels were investigated for their influence on osteoclastogenesis^37^. It was demonstrated that calcite containing xerogels exhibit a higher resorption by osteoclasts than hydroxyapatite containing xerogels. Thus, variation of chemical composition of multiphasic xerogels was shown to be an important tool in tailoring resorption rates. In continuation to this previous study and earlier published studies on silica/collagen xerogels with incorporated calcium phosphate phases^25,26,28^, the present study focuses on the effect of calcite incorporated in silica/collagen xerogels on bioactivity, calcium release, as well as the proliferation and osteogenic differentiation of hMSC. Biphasic xerogels, as well as triphasic xerogels, the latter with either hydroxyapatite or calcite, were prepared. The structure of the multiphasic xerogels was investigated by transmission electron microscopy (TEM), especially regarding the influence of hydroxyapatite and calcite onto the composite microstructure. Bi- and triphasic xerogels were studied for their ion release and bioactivity. The supposed release of calcium ions due to the incorporation of calcite was evaluated related to the support of proliferation and osteogenic differentiation of hMSC. Furthermore, this investigation contributes to the understanding of the remodeling of degradable substitute materials.

## Results

### Structural characterization of multiphasic silica/collagen xerogels

Structural investigations on multiphasic silica/collagen xerogels were performed by using TEM and scanning electron microscopy (SEM). For TEM, thin sections of xerogel granules and for SEM, both thin sections of xerogel granules and xerogel granules themself were used. In TEM analysis, silica was unambiguously detected by energy dispersive X-ray spectroscopy (EDX) signals of silicon (Si) and oxygen (O), hydroxyapatite by signals of calcium (Ca) and phosphorus (P) and calcite by EDX signals of Ca, carbon (C) and O. Characteristic elements for proving collagen are hydrogen (H), C, O and nitrogen (N). Due to the coexistence of C and O in both collagen and the used embedding material, these elements were no unambiguous proof. H could not be detected by EDX because of creating soft X-ray radiation. N occurs in small amounts as aminogroup in collagen but was imprecisely detectable because of proportionally soft X-ray radiation. The proof of collagen fibrils was done morphologically.

**Figure 1:**
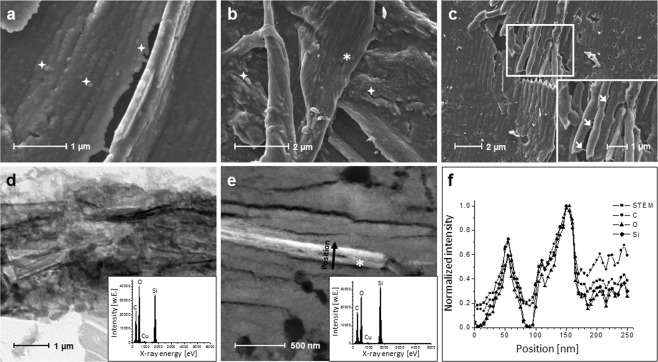
Organization of silica and collagen in multiphasic xerogels (by the example B30). SEM images of agglomerated spherical silica particles (**a**), collagen fibrils arranged in parallel in the silica/collagen matrix, as well as fibril bundle (**b**), and filamentary bondings of low fibrillar collagen organized in between silica agglomerates (**c**, higher magnification of selection in inset). Bright field TEM image of silica/collagen matrix (**d**) and related EDX spectrum (**d**, inset). Dark field TEM image of two collagen fibrils in parallel within silica/collagen matrix (**e**) and related EDX spectrum (**e**, inset). Intensity profile of the line scan (**f**) along the black arrow in (**e**) indicating two silica sheathed collagen fibrils. Agglomerated silica particles were exemplarily indicated with stars, low fibrilar collagen with arrows and collagen fibrils with asterisks.

SEM and TEM images of a B30 thin section are used to characterize organization of the two components silica and collagen occuring in all three xerogel types B30, the hydroxyapatite containing B30H20 and the calcite containing B30CK20. Makrostructure obtained from ultramicrotome preparation brings out details on the composite microstructure. Relevant details are shown in Fig. 1. In SEM images, silica occurred as agglomerates of spherical nanoparticles (Fig. 1a–c, exemplarily indicated with stars). In TEM images, not only Si and O indicating silica but also C occurred (Fig. 1d, Cu belongs to the copper grid). This indication of collagen within silica (silica/collagen matrix) is confirmed morphologically by SEM. Filamentary bondings are organized in between silica particle agglomerates (Fig. 1c, inset, exemplarily indicated with arrows). Moreover, Fig. 1b shows the presence of collagen fibrils (exemplarily indicated with asterisk) parallelly arranged in the silica/collagen matrix, as well as a collagen fibril bundle. TEM investigations on fibrilar morphologies displayed an increase of signal intensity for Si, O and C in direction to the fibrils center of longitudinal EDX line scan as well as decreased intensities in direction to the fibrils edges indicating that the collagen fibrils were sheathed by silica (Fig. 1e,f).

**Figure 2:**
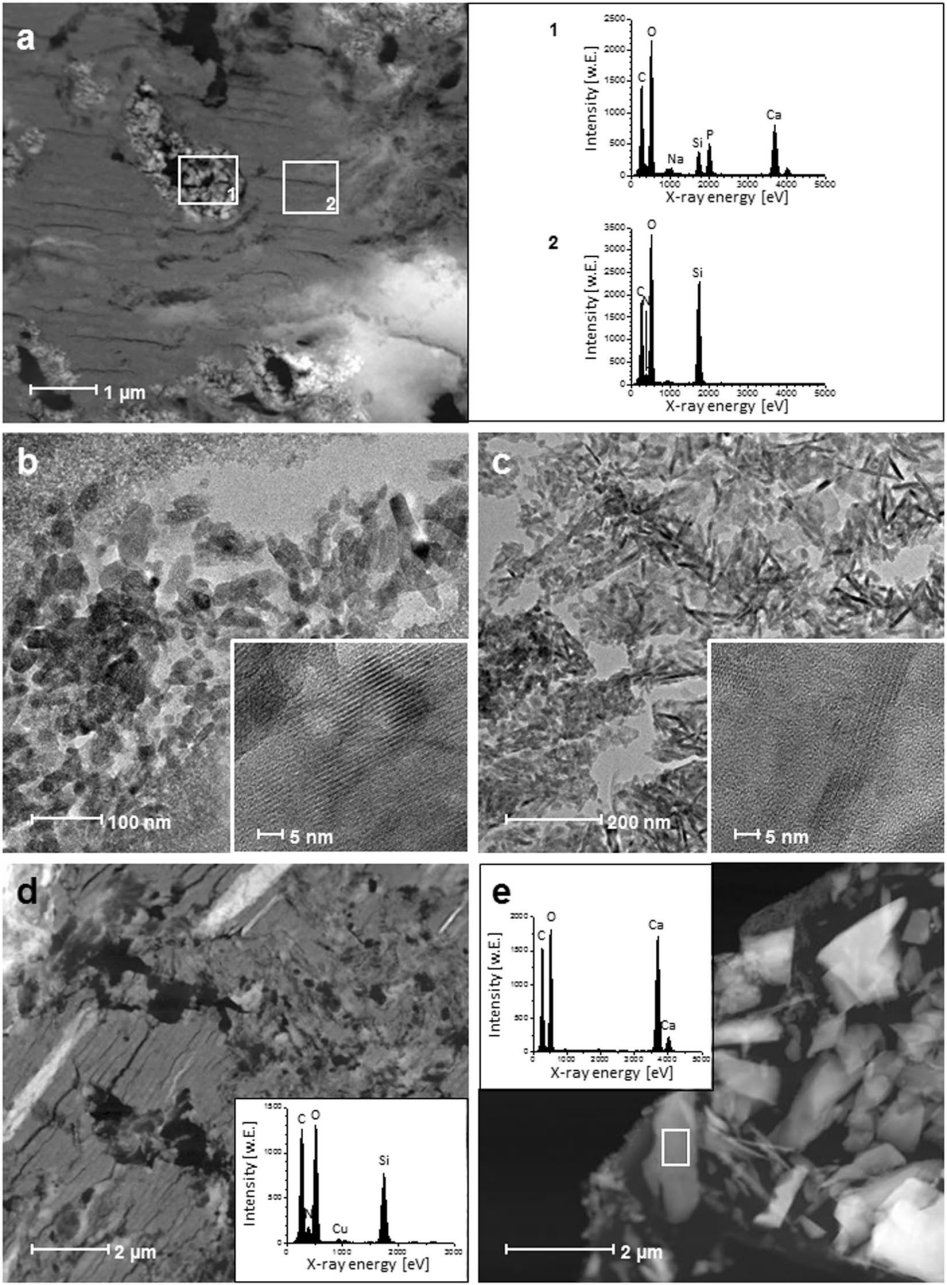
Incorporation of hydroxyapatite or calcite as mineral phase. Dark field TEM image and related EDX spectra of a B30H20 thin section (**a**) with a hydroxyapatite-enriched phase (selection 1, EDX spectrum 1), embedded in the homogeneous silica/collagen matrix (selection 2, EDX spectrum 2). Bight field TEM images of granular (**b**) and rod-shaped (**c**) hydroxyapatite in B30H20 and related high resolution TEM images with lattice layers (**b**,**c** insets). Dark field TEM images and related EDX spectra (insets) of the silica/collagen matrix in B30CK20 (**d**) and the individually available calcite phase (**e**).

Thin sections of B30H20 were characterized by homogeneous regions with embedded granular or rod-shaped structures in micrometer range (Fig. 2a–c). Homegeneous structures (Fig. 2a, selection 2) contained C, O and Si as well as small amounts of N, indicating collagen within silica as shown before in Fig. 1. In granular and rod-shaped structures (Fig. 2a, selection 1), Ca and P as well as small amounts of Na were detected among C, O and Si, indicating silica and collagen between hydroxyapatite particles. The Na signal might belong to remains of the sodium phosphate buffer solution to fibrillate tropocollagen and displays an additional proof for the presence of collagen. Images of granular and rod-shaped structures in high resolution mode displayed visible atom columns, which are visible as alternating bright and dark lattice layers, indicating crystallinity and identifying hydroxyapatite (Fig. 2b,c, insets) in comparison to the amorphous silica mineral phase. Thin sections of B30CK20 exhibited plate-like structures in micrometer range, partially planar, partially rod-shaped. Rods and small platelets consisted of Ca, C and O, indicating calcite (Fig. 2e). Moreover, signals of C, O and Si as well as small amounts of N were detected, indicating the silica/collagen matrix. (Fig. 2d, Cu belongs to the copper grid).

**Figure 3:**
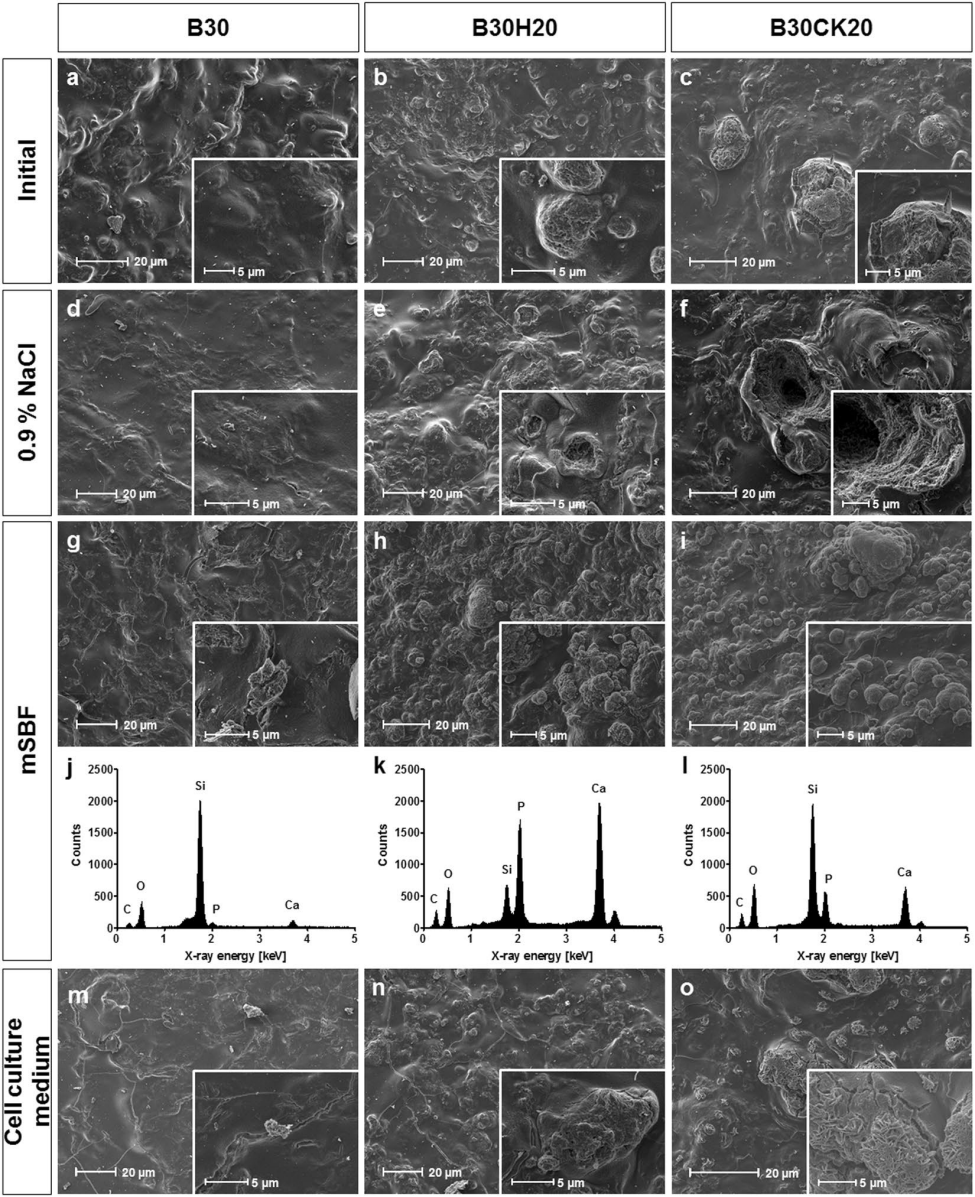
SEM images of xerogel surfaces of B30 (**a**,**d**,**g**,**m**), B30H20 (**b**,**e**,**h**,**n**) and B30CK20 (**c**,**f**,**i**,**o**) before (**a**–**c**) and after incubation in 0.9% NaCl (**d**–**f**), mSBF (**g**–**i**) and cell culture medium (**m**–**o**). EDX spectra are related to precipitations formed on B30 (**j**), B30H20 (**k**) and B30CK20 (**l**) after immersion in mSBF. Xerogel samples were incubated in liquid medium for 21 days. Medium was changed three times a week. Insets represent regions of interest in higher magnification.

A glance view on the composites surface is presented in Fig. 3, which was supplemented by topographical investigations during incubation in different media. Typical surfaces of B30, B30H20 and B30CK20 visualized by SEM represent the silica matrix containing collagen and agglomerated hydroxyapatite or calcite, respectively, when incorporated (Fig. 3a–c). Superficial morphologies of initial xerogel samples consisted of aggregated silica nanoparticles and randomly oriented fibrillar collagen (Fig. 3a). Additionally, the surface of the triphasic xerogel B30H20 exhibited hydroxyapatite particles as agglomerates of 5–20 µm in diameter covered by silica/collagen composite matrix (Fig. 3b). B30CK20 contained mineral agglomerates in ranges of 5–10 µm in diameter covered by silica/collagen composite matrix as well as larger particles with up to 70 µm in diameter partially uncovered by silica/collagen composite matrix (Fig. 3c).

### Studies on bioactivity and calcium ion release

Studies on bioactivity and the release of calcium ions were done by incubating the different xerogel types B30, B30H20 and B30CK20 in physiological salt solution (0.9% NaCl), modified simulates body fluid (mSBF), and cell culture medium. Changes in surface morphology due to the incubation in 0.9% NaCl were visualized using SEM (Fig. 3d–f). For all xerogel types, superficial microcracks increased within 21 days of incubation in 0.9% NaCl. Additionally, surfaces of B30H20 and B30CK20 exhibited macrovoids formed by separation of material from the respective xerogel during incubation. The calcite containing xerogel B30CK20 exhibited macrovoids up to 40 µm in diameter (Fig. 3f). Moreover, channels into the bulk material starting from these voids are visible. In contrast, voids on the surface of the hydroxyapatite containing xerogel B30H20 were smaller with about 10 µm in diameter (Fig. 3e).

The surfaces of different xerogel types after incubation in mSBF are shown in Fig. 3g–i. Highest new superficial mineral formation was observed on B30CK20 compared to B30 and B30H20. B30CK20 exhibited platelet-like crystals widly distributed on the surface of particles of the incorporated calcite phase which were covered before by the silica/collagen matrix (Fig. 3i). Mineral formation on the hydroxyapatite containing B30H20 (Fig. 3h) and the biphasic B30 (Fig. 3g) occurred as smaller agglomerates of platelet-like crystals compared to the calcite containing B30CK20. EDX spectra related to precipitated crystals on xerogel surfaces were shown in Fig. 3j–l. Spectra of different xerogel types after incubation in mSBF exhibited Ca and P peaks indicating that precipitated crystals were composed of calcium phosphate. Moreover, all spectra contain Si and O peaks representing silica from the xerogel substrate. In case of incubated B30 and B30CK20, peaks of Si showed a higher intensity compared to peaks of Ca and P. In case of B30H20, the intensity of Ca and P peaks was higher than the intensity of Si peak.

The surface morphology of B30, B30H20 and B30CK20 after incubation in cell culture medium is presented in Fig. 3m–o. In general, mineral formation was less than after incubation in mSBF. As observed for mSBF, B30CK20 offered extended mineral formation on the surface of particles of the incorporated calcite phase compared to B30 and B30H20. In contrast to the incubation in mSBF, incubation in cell culture medium caused elongated platelets of crystals (Fig. 3o). Morphology of formed mineral on B30H20 (Fig. 3n) and B30 (Fig. 3m) was similar to that precipitated while incubated in mSBF but the amount was less.

**Figure 4:**
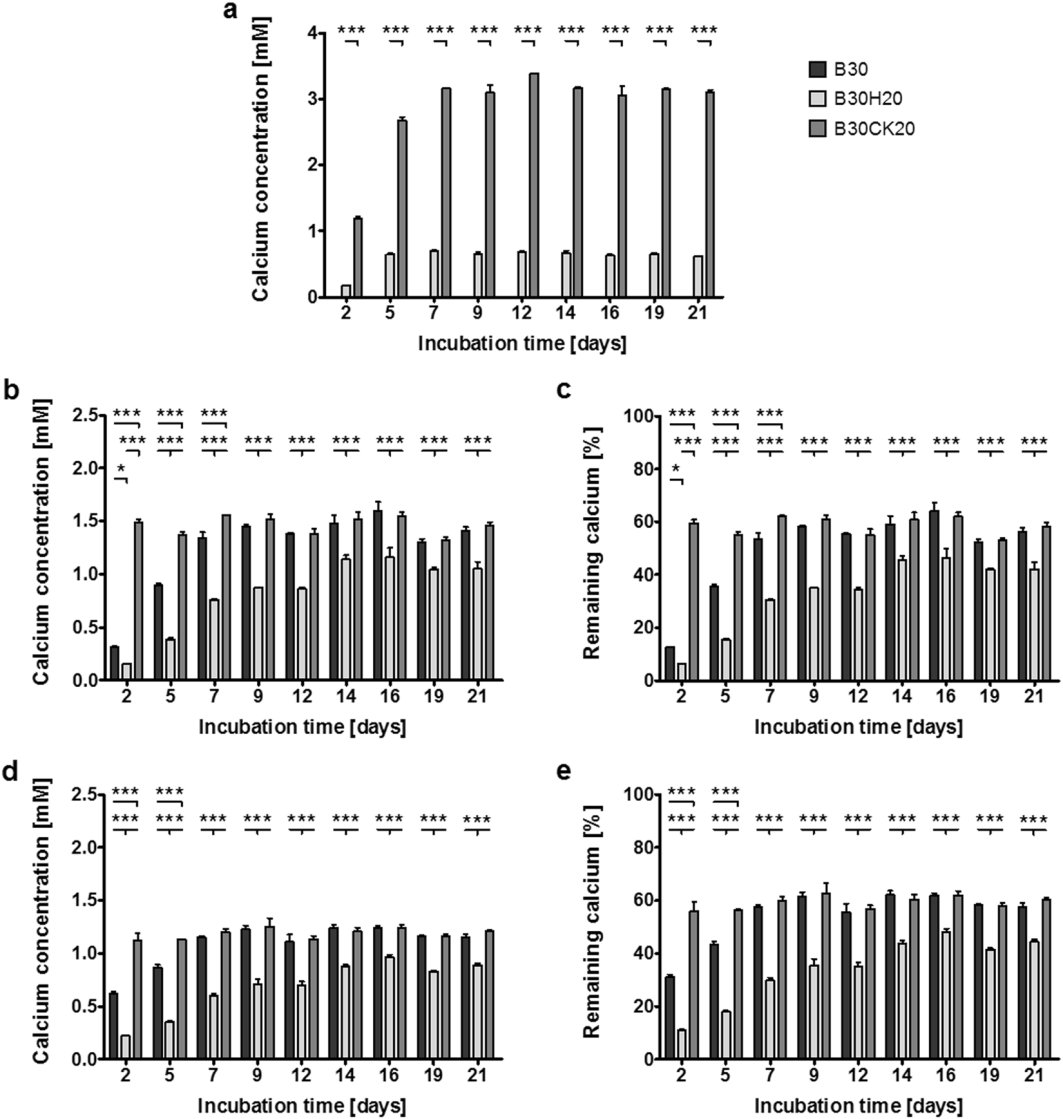
Calcium in 0.9% NaCl solution (**a**), mSBF (**b**,**c**) and cell culture medium (**d**,**e**) after incubation of B30, B30H20 and B30CK20, respectively, as absolute concentrations (**a**,**b**,**d**) and as concentrations relative to the initial concentration (**c**,**e**). Xerogel samples were incubated in liquid medium for 21 days. Medium was changed three times a week. Calcium concentrations are representative for each 2–3 days static incubation until medium refreshment. *p < 0.05, **p < 0.01, ***p < 0.001.

Measuring the ion concentrations during composite incubation in 0.9% NaCl solution revealed for the biphasic silica/collagen xerogel B30 no detectable calcium concentration after immersion for 21 days, as expected (Fig. 4a). Over the same incubation time, both hydroxyapatite containing B30H20 and calcite containing B30CK20 released calcium ions into 0.9% NaCl solution with each medium refreshment. Both xerogel types caused an increase in released calcium ions within the first 7 days including 3 medium changes. In the following, the calcium concentration was constant. Amount of released calcium ions was approximately 4.5 times higher for B30CK20 (3.2 mM) than for B30H20 (0.7 mM).

The two different calcium and phosphate containing solutions, mSBF and cell culture medium, were used for investigations on bioactivity. The reduction of the solutions calcium concentration (calcium consumption) as well as mineral formation on the sample surface, as shown before, were consulted for characterization of bioactivity. Ion concentrations of mSBF are equal to those of blood, except hydrogen carbonate. Calcium concentration in mSBF was adjusted to 2.5 mM. Used cell culture medium with added fetal calf serum (FCS) contains amino acids and proteins in contrast to mSBF. Calcium concentration in cell culture medium (including FCS) amount to approximately 2 mM. During incubation of the xerogel samples, media were completely changed three times a week. Calcium concentrations in the supernatants obtained in the process were detected (Fig. 4).

After immersion in mSBF, all xerogel types caused a reduced calcium concentration of their environment over 3 weeks (Fig. 4b,c), being maximal during the first week. Highest reduction of calcium concentration in mSBF was observed for B30H20, especially during the first two days of incubation (0.2 mM, 8% of initial calcium). Lowest initial reduction of calcium concentration in mSBF was obtained by the incubation of B30CK20 (1.5 mM, 60% of initial calcium). During last two weeks of incubation in mSBF, calcium concentration obtained by B30 and B30CK20 was similar (1.3–1.6 mM, 52–64% of initial calcium).

The incubation of all xerogel types in cell culture medium caused reduced calcium concentrations over 21 days (Fig. 4d,e). As shown for mSBF, calcium concentration in cell culture medium obtained for B30H20 was lowest (0.2–1.0 mM, 10–50% of initial calcium). B30CK20 caused a constant calcium concentration in cell culture medium over time (1.2 mM, 60% of initial calcium). Calcium concentration caused by B30 in cell culture medium was between the measured values of B30CK20 and B30H20 (0.6–1.2 mM, 30–60% of initial calcium).

### Proliferation and osteogenic differentiation of hMSC

Xerogel types B30, B30H20 and B30CK20 were characterized concerning cell attachment, proliferation and osteogenic differentiation of hMSC. Moreover, the influence of xerogels degradation products, as well as bioactivity-dependent ion release and reprecipitation alone was studied using indirect cell culture and the additional influence of direct cell-material contact was characterized using direct cell culture. Cells were cultured without osteogenic stimuli (Os−) until day 3. Henceforward, they were either continued culturing as non-induced cells or were stimulated with osteogenic supplements (Os+) until day 28.

**Figure 5:**
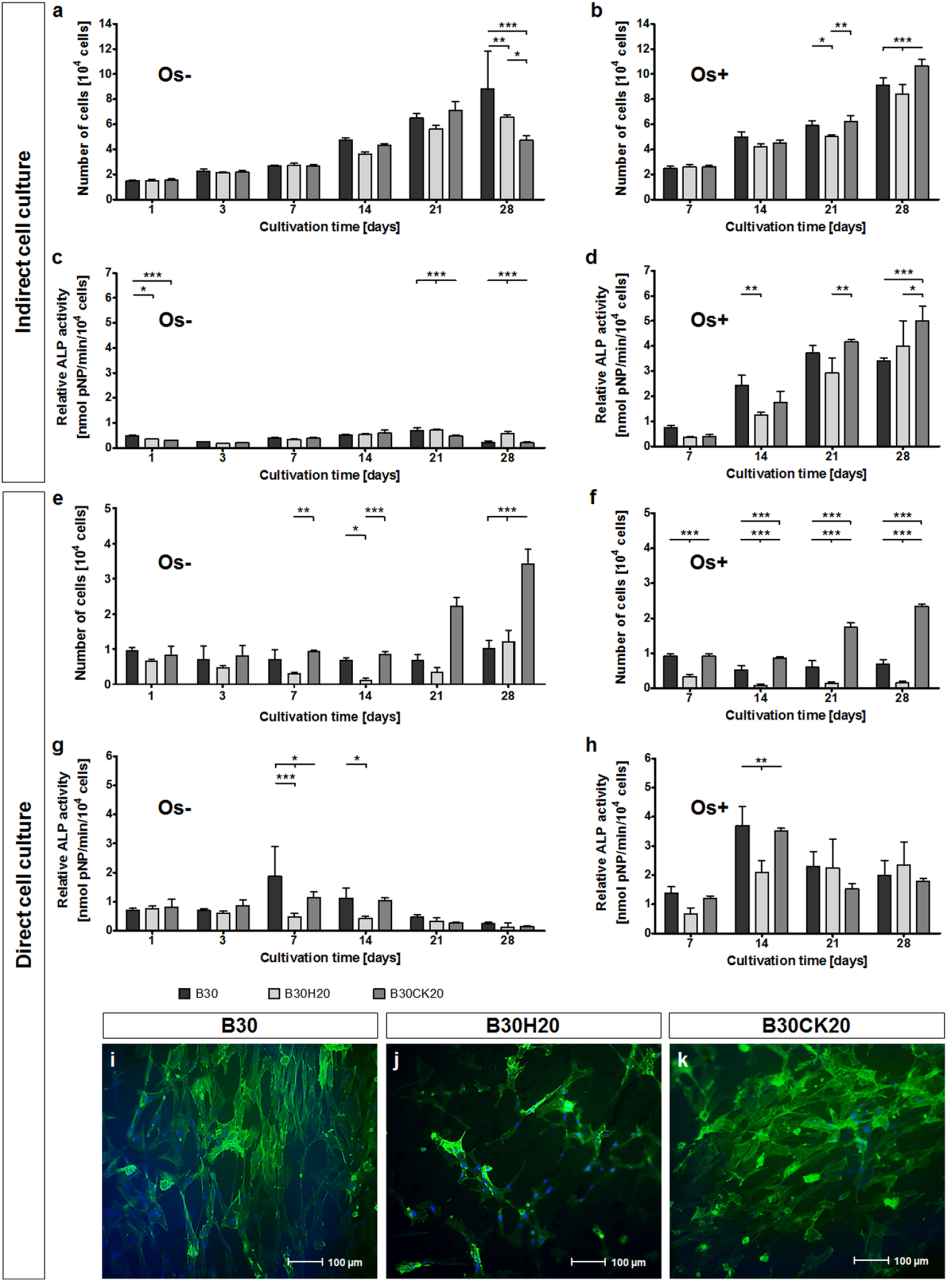
Proliferation (**a**,**b**,**e**,**f**) and osteogenic differentiation (**c**,**d**,**g**,**h**) of hMSC cultured in presence (indirect cell culture, **a**–**d**) and on the surface (direct cell culture, **e**–**h**) of B30, B30H20 and B30CK20. Attachment of hMSC on the surface of B30 (**i**), B30H20 (**j**) and B30CK20 (**k**), 24 h after seeding. Cells were seeded at 1.7 × 10^4^ per 24-well and 2 × 10^4^ per xerogel, respectively and were treated with 50 µM ascorbate on the first day of adherence (Os−; **a**,**c**,**e**,**g**). For osteogenic differentiation (Os+; **b**,**d**,**f**,**h**), hMSC were treated with 10 nM dexamethasone, 5 mM β-glycerophosphate and 50 µM ascorbate by day 3. Number of cells and ALP activity were measured from cell lysates by biochemical assays. *p < 0.05, **p < 0.01, ***p < 0.001. In fluorescence microscopic images, nuclei and F-actin were stained in blue and green, respectively.

For indirect cell culture, number of cells cultured in presence of xerogels was nearly unaffected by the different types until day 21 for Os− cells and day 14 for Os+ cells (Fig. 5a,b). Number of Os+ cells cultured in presence of the hydroxyapatite containing B30H20 was below that of the pure B30 and the calcite containing B30CK20, especially on day 21 und day 28. A small difference in proliferation of Os+ cells cultured in presence of B30 and B30CK20 is given on day 28 with an increased cell number in presence of B30CK20. Osteogenic differentiation, represented by ALP activity, increased over time for the cultivation of Os+ cells in presence of B30H20 and B30CK20 with maximum on day 28 (Fig. 5c,d). For B30, ALP activity increased until day 21 and stayed constant further on. Generally, ALP activity of Os+ cells in presence of the hydroxyapatite containing B30H20 was below that of the calcite containing B30CK20. ALP activity of Os− cells in presence of xerogels was nearly unaffected.

After 24 h of direct cell-material contact, cell attachment was slightly decreased for the hydroxyapatite containing B30H20 compared to B30 and B30CK20. These differences in cell attachment determined by biochemical method were confirmed by fluorescence microscopy (Fig. 5i,j,k). Moreover, fluorescence images show a more elongated morphology of hMSC on both B30 and B30CK20 compared to hMSC on B30H20. In addition, cell number on B30H20 was constantly below that on B30 and B30CK20 until day 21 for Os− cells and until day 28 for Os+ cells (Fig. 5e,f). Proliferation of non-induced Os− as well as induced Os+ cells was significantly increased for B30CK20 between day 21 and day 28 in comparison to B30 and B30H20. Osteogenic differentiation of cells with direct material contact was preferably observed for both B30 and B30CK20. Maximum of ALP activity occurred around day 14 for Os+ cells on B30 and B30CK20, afterwards ALP activity decreased and stayed constant further on (Fig. 5h). ALP activity of Os+ cells on B30H20 raised on day 14 and stayed constant until day 28. Maximum ALP activity of B30H20 was lower compared to B30 and B30CK20, but all three xerogel types were almost at the same level for day 21 and day 28. Furthermore, Os− cells on B30 and B30CK20 showed a small increase in ALP activity around day 7 and day 14 compared to the remaining days (Fig. 5g). ALP activity of Os− cells on B30H20 did not rise.

Comparing proliferation of cells from indirect and direct cell culture, increase in cell number for B30CK20, as well as differences between the three xerogel types are more distinct in case of direct cell-material contact. Furthermore, an increase in ALP activity was observed in the absence of osteogenic supplements (Os−) for direct cell culture.

## Discussion

In our previous studies, biphasic and triphasic silica/collagen xerogels were developed and investigated, the latter containing an additional calcium phosphate phase. Silica/collagen xerogels were shown to be bioactive by binding calcium and phosphate ions from their liquid environment to their surface, resulting in the formation of an apatite layer^25,26^. We found that bioactivity of the multiphasic silica/collagen xerogels displays a tool for manipulating the ratio of osteoblasts to osteoclasts^28^. In this study, we focus on increasing the calcium concentration in the liquid environment of multiphasic xerogels. For this, the calcium ion release must be greater than the precipitation of mineral at its surface, which is all the more important in the treatment of bone fractures with a previous disease, e.g. osteoporosis. In this particular case, the recruitment of osteoblasts and their precursor cells, as well as their proliferation and osteoblastic differentiation should be stimulated, which is why the net release of calcium ions from the substitute materials is highly desirable^16^. The solution to that challenge could be the incorporation of commercially available calcite as the third phase in silica/collagen xerogels.

Silica/collagen xerogels in the form as biphasic (B30) and triphasic composites with the latter containing either hydroxyapatite (B30H20) or calcite (B30CK20) were analyzed for their structure using TEM and SEM. As demonstrated by SEM, aggregated silica nanoparticles in B30 acted as matrix for collagen fibrils as reinforcing structural element. These findings were confirmed and supplemented by TEM. Figure 1e,f show the parallel arrangement of collagen fibrils along their longitudinal direction, which are sheathed by silica, indicating that collagen acts as template for silica nanoparticles previously found by Eglin *et al*.^38^. Moreover, by analyzing bright field TEM images of biphasic xerogels B30 by EDX, signals attributed to collagen were found homogenously distributed within the silica matrix phase. That indicates low fibrillar collagen acting as template for silica. Similar structures were found in human bone, where collagen represents the template for calcium phosphate mineralization. Between the mineralized collagen fibrils within these structures, sacrificial bonds and hidden length are evident that dissipate energy at crack initiation, slowing or stopping their propagation^39^.

Concluding from the results of the present study, biphasic silica/collagen xerogels B30 exhibited two structural levels, namely parallel arranged collagen fibrils in a silica-enriched phase that in turn contains low fibrillar collagen (Fig. 1). Three structural levels were observed for both hydroxyapatite and calcite containing silica/collagen xerogels (B30H20 and B30CK20) (Fig. 2). As shown for biphasic xerogels B30, triphasic xerogels also exhibit low fibrillar collagen acting as template for silica and collagen fibrils, which occured within the silica-enriched matrix. For hydroxyapatite containing xerogels B30H20, agglomerates of hydroxyapatite particles occurred homogenously distributed within the silica-enriched phase. This hydroxyapatite-enriched phase consisted of hydroxyapatite particle agglomerates being present next to silica and low fibrillar collagen that stabilizes the composite. Hydroxyapatite in triphasic xerogels was identified in both granular and rod-shaped morphology although it was initially incorporated as granular particles only. A possible explanation for the presence of rod-shaped hydroxyapatite can be found in the local acidification of hydroxyapatite by silicic acid followed by nucleation in rod-like morphology during xerogel preparation – a process of solution and reprecipitation. In contrast to hydroxyapatite, calcite in B30CK20 is found isolated within the silica-enriched phase because of its larger particle size. The structural levels of multiphasic xerogels and the incorporation manner of either hydroxyapatite or calcite were identified to affect calcium release as discussed later.

Studies on bioactivity of xerogels, as well as on calcium ion release were performed by detecting the calcium content after immersion of xerogels in different media for a period of 21 days including regular medium changes. Medium refreshment was used to simulate conditions comparable to cell culture, as well as to prevent gradual saturation of medium, which affects ion exchange over the performed long-term investigation. To study calcium ion release from xerogels only, physiological salt solution (0.9% NaCl) as calcium-free reference medium was used. The amounts of released calcium ions from both hydroxyapatite containing B30H20 and calcite containing B30CK20 xerogels are nearly constant over time. Calcite exhibits a higher standard solubility product than hydroxyapatite resulting in a markedly higher amount of released calcium ions for B30CK20. Moreover, crystal size and thus the resulting specific surface in contact to the liquid affect the release kinetics. The different incorporation manners of hydroxyapatite and calcite, respectively, might contribute to the different release kinetics as well. The accessibility for water molecules to the calcite crystal surface in B30CK20 is enhanced compared to that to the hydroxyapatite surface in B30H20 (Fig. 3h,i). Furthermore, the dissolution of calcite in B30CK20 is not interfered by the silica-enriched phase, as it is the case for hydroxyapatite agglomerates in B30H20. As a result, larger macrovoids including channels into the interior of the material are present. The release of calcium ions is overlain by the bioactivity of multiphasic silica/collagen xerogels. Bioactivity was studied by the incubation in calcium and phosphate containing solutions with or without amino acids and proteins (cell culture medium and mSBF, respectively) followed by SEM investigations. The overlay of calcium ion release and reprecipitation due to the samples bioactivity is most apparent for the calcite containing B30CK20. The calcium ion releasing B30CK20 effected almost the same calcium concentration in mSBF as the calcium-free B30. However, superficial mineral layer on B30CK20 was most distinct in comparison to B30 and B30H20. As previously described for silica/collagen xerogels at all^25^, silanol groups (Si-OH) on the surface of silica nanoparticles induce apatite nucleation in SBF^40^. Among adsorption of calcium ions and phosphate ions resulting in heterogeneous nucleation, chemical reactions between silanols and phosphate ions complex both calcium and phosphate ions^41^. These precipitates from mSBF on the xerogels surface were investigated by semi-quantitative EDX measurements (Fig. 3j–l). For all xerogel types, precipitates were identified to be calcium phosphate. In case of the hydroxyapatite containing B30H20, high intensity of Ca and P peaks in EDX spectrum of the precipitates not exclusively belong to the superficially formed calcium phosphate layer but also to the embedded hydroxyapatite phase in the bulk of the xerogel. Moreover, the Ca peak in the B30CK20 spectrum may also belong to the incorporated calcite in the xerogel. In general, all spectra contain information from the silica/collagen bulk material, which is represented by peaks of Si and O. Calcite or hydroxyapatite crystals in silica/collagen xerogels might also act as nuclei for heterogeneous calcium phosphate nucleation resulting in an enhanced nucleation in comparison to the biphasic B30. The lower amount of mineral formation at B30H20 compared to B30CK20 can be explained by the smaller size of mineral agglomerates on the surface of B30H20 and consequently fewer nuclei for heterogeneous nucleation. The growth of calcium phosphate crystals on calcite substrates was also observed by Naidu *et al*.^42^ and Cruz *et al*.^43^. Changed morphology of superficial mineral crystals was observed comparing mSBF and cell culture medium. In general, mineral layers on the different xerogel types were characterized by less nuclei when immersed in cell culture medium compared to mSBF. Several studies showed that proteins inhibited or slowed down superficial apatite formation^44–48^. Lee *et al*. published that FCS proteins in cell culture relevant concentrations inhibit apatite precipitation on hydroxyapatite surfaces because they adsorb on the surface and occupy sites for crystal nucleation^44^. Juhasz *et al*., who immersed β-tricalcium phosphate as well as hydroxyapatite in 100% human blood serum, hypothesized a dual effect of the biological compounds on the crystal nucleation process. The ability of proteins to chelate calcium ions decreases the amount of free calcium ions in the solution preventing apatite nucleation. Conversely, absorbed proteins can enhance heterogeneous nucleation due to changes in surface charges or creation of nucleation sites^46^.

The important role that extracellular calcium plays in bone remodeling results, among other things, from the activation of the CaSR^15,17,23^, and by voltage dependent Ca^2+^-channels, which are important for the maintenance of intracellular calcium homeostasis^17,49^. In the present study, cell culture experiments using hMSC were performed in presence or directly on the top of different xerogel types. By the help of indirect cell culture, the effects of degradation products as well as ambient ion conditions on hMSC/osteoblasts are evaluated, which in turn are controlled by both ion release and bioactivity-dependent reprecipitation. In addition to the effects in indirect cell culture, the close interaction of hMSC/osteoblasts with the material surface, also including surface morphology, plays an important role in direct cell culture. For indirect cell culture, cells were grown on polystyrene surfaces of tissue culture plates and thus having optimal conditions in attachment, spreading and proliferation, only affected by degradation products. Culture conditions on xerogel surfaces are more complex than on polystyrene. Figure 3a–c demonstrates that xerogel surfaces are not flat. Surface related changes in culture conditions like roughness, morphology, wettability etc. might be responsible for differences in the number of cells and their proliferation behavior compared to indirect cell culture. Despite of differences in the absolute values, number of cells indirectly and directly cultured with and without osteogenic supplements was increased for B30CK20 and B30 compared to B30H20. In earlier studies, it has been reported by other authors that the proliferation of osteoblasts, as well as their precursor cells is enhanced with increasing extracellular calcium concentration^18,19^. Proliferation of rat MSC on polystyrene was shown to be promoted in dose-dependent manner for calcium concentrations up to 10 mM after 3 days of culture^18^. Similar results were observed for porcine MSC on polystyrene stimulated with calcium concentrations of 1–6 mM for 5 days^19^. In the same study, Ye *et al*. showed the association of the enhanced proliferation with the activation of the plasma membrane CaSR and the elevation of intracellular calcium concentration. Furthermore, our own previous study showed reduced proliferation of hMSC on silica/collagen xerogels containing different calcium phosphate phases, which caused a reduced extracellular calcium concentration^28^. For B30H20, causing a low environmental calcium concentration, our results are in agreement to all these studies. Interestingly, an almost equal extracellular calcium concentration in cell culture medium was detected for B30 and B30CK20, while B30CK20 showed best results in proliferation. Considering the more distinct increase in cell number for direct cell culture compared to the indirect one, this may occur because of the direct contact of cells to the calcium ion releasing B30CK20 and thus a locally enhanced calcium concentration. In addition to calcium ions, carbonate is released from B30CK20 which contains 20% calcite. Pieters *et al*. studied the influence of apatites containing different contents of carbonate on the behavior of a mouse osteoblast precursor cell line (MC3T3-E1)^50^. They reported the support of cell adhesion and proliferation for apatites with a higher than 11% carbonate content. In recent literature, osteogenic differentiation, among adhesion and proliferation, is reported to be stimulated by enhanced extracellular calcium concentrations^17,20,22,30,51,52^. In the present study, ALP activity of cells cultured in presence or with direct contact to both B30 and B30CK20 was elevated in comparison to that of cells cultured on B30H20. For direct cell culture, that increase was observed for cells both treated and not treated with osteogenic supplements (Os− and Os+ cells). Thus, osteogenic differentiation was increased with increased extracellular calcium concentrations, even in the absence of osteogenic supplements. Maeno *et al*. observed an increase in the number of mouse primary osteoblasts positively stained for ALP, an early marker for osteogenic differentiation, for extracellular calcium concentrations up to 4 mM^20^. Moreover, the expression of osteocalcin, a late marker for osteogenic differentiation, was enhanced for 6–15 mM extracellular calcium. Yamauchi *et al*. demonstrated an increase in osteogenic differentiation of the mouse osteoblast precursor cell line MC3T3-E1 in terms of mineralization with an increase in calcium concentration (1.8–3.8 mM)^22^. Wagner *et al*. published that increased calcium concentrations (1.8–20 mM) are correlated with an increase in the expression of connexin 43, the gap-junction forming protein, and in bone sialoprotein, a further osteogenic marker, as well as in matrix mineralization. Moreover, they suggested that regulatory effects at the hemichannel level - that form the pore for a gap junction - are calcium-dependent^30^.

## Conclusions

Multiphasic silica/collagen xerogels are biomaterials, which allow to manipulate cellular processes. The present study demonstrates that calcite incorporated into silica/collagen xerogels can be used for calcium delivery. Despite of similar calcium concentration in mSBF and cell culture medium for B30CK20 compared to the biphasic silica/collagen xerogel B30, proliferation of hMSC was enhanced, which is caused by the direct contact of cells to the calcium ion releasing bone substitute material. In general, by varying the composition of the silica/collagen xerogel - as pure one, with hydroxyapatite or with calcite - their bioactivity varies as well that in turn affects the related calcium concentration of the surrounding liquid and thus cellular reaction. Therefore, multiphasic silica/collagen xerogels can be considered to mediate osteoblast proliferation and differentiation to a desired extent. In addition to tailoring osteoclastic resorption by the xerogels properties, which was shown before, mediation of the osteogenic differentiation might be a key regulator of the osteoblast/osteoclast ratio during the remodeling of bone and the bone substitute material as well. For prospective application *in vivo* we can envision diverse possibilities like an application as pre-shaped monolithic material, as a granular filler material or as granular component to a collagenous scaffold.

## Materials and Methods

### Preparation of xerogels

Bovine tropocollagen type I (GfN, Germany) was dialysed (MWCO 12–14 kDa, Roth, Germany) against deionized water, fibrillated in 30 mM neutral sodium phosphate buffer solution and lyophilized (Christ Apha1-4 laboratory freeze-dryer, Germany). A homogenous collagen suspension of 30 mg/mL was formed by resuspension in 0.05 M Tris/HCl pH 8 (Roth, Germany), as described previously^26^. Amounts of hydroxyapatite (Ca_5_(PO_4_)_3_(OH), Merck, Germany) or calcite (CaCO_3_, Merck, Germany) as mineral phase were calculated according to Heinemann *et al*.^26^ and added to the collagen suspension followed by rigorous stirring to prepare triphasic xerogels. For preparation of silicic acid, an aqueous tetraethoxysilane solution (TEOS, 99%, Sigma, Germany; molar ratio TEOS/water = 1/4) was hydrolyzed under acidic conditions (0.01 M HCl). By vigorous stirring of calculated amounts of collagen suspension, silicic acid and mineral phase, according to Heinemann *et al*.^26^, using a vortex mixer, hydrogels with the final biphasic composition of 30% bovine collagen and 70% silica (B30) and the final triphasic compositions 30% bovine collagen, 50% silica and 20% hydroxyapatite (B30H20) or calcite (B30CK20) were formed. After stabilization for 3 days, hydrogels were dried until mass constancy in a climate chamber (Espec SH-221, Japan) at 37 °C and 95% relative humidity followed by a climate ramp to achieve ambient conditions. Resulting xerogels were present in form of stable monoliths (6 mm in diameter, 3 mm in height). Xerogels were gamma-sterilized at 25 kGy (BBF Sterilisation Service GmbH, Germany).

### Studies on bioactivity and calcium ion release

Biphasic B30 as well as triphasic B30H20 and B30CK20 monolithic xerogels were incubated in different media, such as 0.9% NaCl solution (Sigma-Aldrich, Germany), mSBF according to Oyane *et al*.^53^ and cell culture medium consisting of modified eagles medium (α-MEM, Biochrom, Germany) containing 10% FCS (Biochrom, Germany), 2 mM glutamine (Biochrom, Germany), 100 U/mL penicillin and 100 µg/mL streptomycin (Biochrom, Germany). In a humidified atmosphere at 37 °C and 8.5% CO_2_, samples were separately immersed in 1.2 mL medium. Media were changed completely three times a week. Supernatants were stored at −80 °C. For surface investigations, xerogels were removed from media, rinsed three times in destilled water and dried at 37 °C.

### Cell culture experiments

The hMSC used in this study were isolated from bone marrow aspirates and kindly provided by Prof. Bornhäuser and co-workers, Medical Clinic I, Dresden University Hospital. The cells were expanded in Dulbecco’s modified Eagle’s medium (DMEM, Biochrom, Germany), supplemented with 10% FCS, 2 mM glutamine, 100 U/mL penicillin and 100 µg/mL streptomycin. For indirect cell culture, 24-well polystyrene culture plates (TPP, Switzerland) were seeded with 1.7 × 10^4^ hMSC in passage 5 contained in 800 µL cell culture medium. For equilibration, monolithic xerogels were incubated overnight in 1 mL α-MEM containing 10% FCS. The next day, xerogel samples (in modified ThinCerts, Greiner, Germany) were transferred to adherent cells on their initial day and medium was changed to cell culture medium supplemented with 50 µM ascorbate (Sigma-Aldrich, Germany) (Os−). For direct cell culture, monolithic xerogels were equilibrated overnight in 500 µL α-MEM containing 10% FCS. The next day, hMSC were seeded at 2 × 10^4^ in passage 5 per xerogel in 450 µL cell culture medium. Cells were additionally treated with 50 µM ascorbate by day 1 (Os−). For osteogenic differentiation (Os+), hMSC were treated with 10 nM dexamethasone (Sigma-Aldrich, Germany), 5 mM β-glycerophosphate (Sigma-Aldrich, Germany) and 50 µM ascorbate by day 3. Medium was changed three times a week. For biochemical analysis, cells were frozen at −80 °C after washing twice with phosphate buffered saline (PBS). For microscopic investigations on cell adhesion, attached cells on xerogel monoliths were fixed with 3.7% formaldehyde (VWR) in PBS after washing twice with PBS. Samples were stored in 0.37% formaldehyde in PBS at 4 °C until usage.

### Biochemical analysis

Frozen samples from cell culture were thawed on ice for 20 min. Cell lysis was performed with 1% Triton X-100 (Sigma-Aldrich, Germany) in PBS for 60 min with additional sonication for 10 min. Afterwards, activity of lactate dehydrogenase (LDH) and of ALP were detected from lysates.

#### LDH activity

Substrate solution of LDH Cytotoxicity Detection Kit (Takara, France) was prepared according to the manufacturer information. Cell lysates (50 µL) were incubated with 50 µL substrate solution for 15 min in the dark. Reaction was stopped using 50 µL 0.5 M HCl (Roth, Germany). Absorbance was measured at 492 nm (Infinite® M200Pro, Tecan, Switzerland). LDH activity was correlated with the number of cells, by using a calibration curve of cell lysates with defined numbers of cells.

#### ALP activity

Substrate solution (100 µL) consisting of 5.4 mM 4-nitrophenylphosphate disodium salt (Sigma-Aldrich, Germany) in substrate buffer containing 100 mM diethanolamin (Sigma-Aldrich, Germany), 1 mM magnesium chloride (Sigma-Aldrich, Germany) and 0.1% Triton X-100 (Sigma-Aldrich, Germany) adjusted to pH9.8 was added to 25 µL cell lysates. After incubation for 30 min at 37 °C, reaction was stopped with 50 µL 0.5 M NaOH (Roth, Germany). Absorbance was measured at 405 nm (Infinite® M200Pro, Tecan, Switzerland). Different concentrations of p-nitrophenol (pNP, Sigma-Aldrich, Germany) in substrate buffer were used as a calibration curve. ALP activity was normalized with respect to the number of cells detected via LDH activity.

### Calcium concentration

Calcium concentrations in the different incubation media were quantified by using the complexometric method Fluitest^®^ CA CPC (Analyticon, Germany). Supernatants were thawed. According to the manufacturer information, both reagents of the kit were mixed. For quantification, 300 µL of this mixture were added to 10 µL supernatant, incubated for 10 min, then the absorbance was determined at 570 nm (Infinite® M200Pro, Tecan, Switzerland).

### TEM

Xerogel monoliths were powdered to granules by using a mixer mill (MM 400, Retsch, Germany). Granules of 250–425 µm in diameter were first infiltrated with a mixture of propylene oxide (Merck, Germany) and araldite (Agar Scientific, United Kingdom) in 3:1 ratio for 4 h, followed by a 1:1 ratio for 12 h and a 1:3 ratio for 4 h, ending up in pure araldite for 4 h. The embedding was performed in araldite for 12 h. Remaining gas was removed by applying vacuum. For hardening, embedded samples were stored at 60 °C for 3 days. The preparation of ultra-thin sections were performed on the ultramicrotome EM Trim (Leica Microsystems, Germany). This sections were put on copper grids and characterized by analytical TEM using a Tecnai F30 (FEI) with an accelerating voltage of 300 kV. Bright field and dark field images were made and samples were investigated by EDX. EDX was applied as a semi-quantitative method for standardless analysis of the amounts of Ca, C, N, O, P and Si.

### SEM

Monolithic xerogel samples from bioactivity and calcium release studies were washed three times with deionized water and dried at 37 °C. For structural investigations, thin sections of embedded xerogel granules from TEM investigations or xerogel granules only were used. Samples on aluminum stubs were coated with C and characterized by an ESEM XL 30 (Philips, Germany) under high vacuum working at 3 kV and detecting secondary electrons. EDX was performed as a semi-quantitative method for standardless analysis of the amounts of Ca, C, O, P and Si.

### Fluorescence staining and fluorescence microscopy

Prior to fluorescence staining, fixed cells were permeabilized for 3 min using 0.2% Triton X-100 in PBS. After washing with PBS, 1% BSA in PBS for 30 min was used to block binding of dyes to nonspecific binding sites. Staining of nucleus was performed by using 20 ng/mL 4′,6-diamidin-2-phenylindol (DAPI, Invitrogen, USA) and staining of F-actin by using 20 µL/ml Alexa Fluor 488® phalloidin (Invitrogen, USA) diluted in 1% BSA in PBS for 60 min protecting from light. Samples were washed several times with PBS. An upright Axioscope 2 FS mot microscope (Zeiss, Germany) was used for fluorescence microscopy.

### Statistics

All degradation and calcium release studies as well as cell culture experiments were performed in triplicates. The results were expressed as means ± standard deviation. Statistical significance was evaluated by analysis of variance (two-way ANOVA, Bonferroni posttest, GraphPad Prism) with p values less than 0.05 (*, significant), 0.01 (**, very significant) and 0.001 (***, highly significant).
